# Predator-mediated diversity of stream fish assemblages in a boreal river basin, China

**DOI:** 10.1038/s41598-023-27854-3

**Published:** 2023-01-11

**Authors:** Jinrui Zhang, Haitao Yang, Mengdi Ma, Tongxiao Pu, Xuwang Yin

**Affiliations:** 1 Liaoning Provincial Key Laboratory for Hydrobiology, College of Fisheries and Life Science, Dalian Ocean University, Dalian, 116023 China; 2 Institute of Remote Sensing and Geographic Information System, School of Earth and Space Sciences, Peking University, Beijing, 100871 China

**Keywords:** Ecology, Biodiversity, Community ecology

## Abstract

Predator–prey interactions are critical for understanding species composition and community assembly; however, there is still limited research on whether and how the prey species composition or community assembly in natural communities are mediated by predators. To address this question, we performed a field investigation to examine the influence of the presence of *Lutra lutra* on the diversity of fish communities of the Hunchun River Basin, Jilin Province, China. Our results indicate that *L*. *lutra*, as a potential umbrella species and generalist predator in the stream ecosystem, promotes the coexistence of a vast variety of fish taxa, which emphasizes the importance of top-down control in the ecological community. We suggest that *L*. *lutra* regulates the fish community assembly likely through the stochastic process. Although this was a pilot study regarding predator–prey interactions, the results highlight the effects of predators on the prey community assembly, and emphasize the role of predators on the maintenance of biodiversity and ecosystem function. Future conservation decisions involving ecosystem biodiversity should require the inclusion of predation intensity. The inclusion of scientific research and protection of umbrella species would thus constitute an additional and important step in biodiversity conservation.

## Introduction

Predator–prey interactions are critical for understanding species composition and community assembly^1,2^, both of which are vital mechanisms for biodiversity maintenance^3–5^. The bottom-up control of prey is an essential resource constraint that influences the diversity of prey species and predator^6,7^; however, top-down regulation theories may fare better in grasping this issue^8–11^. Prey species diversity is controlled from the top-downward due to predation, which promotes diversification or coexistence among prey species by impacting prey species niche differentiation^12^. Several previous studies of laboratory microcosms have verified the role of predators in structuring the assembly and diversity of prey communities^13–15^. Nevertheless, whether and how predators mediate prey species composition or community assembly in natural communities remains unclear.

The community assembly of species is a key question in community ecology and is influenced by a combination of stochastic and deterministic processes^16–19^. Predators can affect the patterns of prey coexistence, relative abundance, and diversity, therefore also influencing the relative importance of stochastic versus deterministic processes in the prey community assembly^13,20^. However, an assessment of this effect is a major challenge because the degree of predator-mediated effects is poorly known. Predators can shape the prey species composition through both consumptive effects (CEs) (i.e., prey removal by predation) and non-consumptive effects (NCEs) (i.e., prey response driven by predation risk)^21,22^, which may shift the coexistence or community assembly of prey species^23^.

The type of predator also plays a key role in the community assembly of prey species. Generalist predators are predicted to reduce *α*-diversity (abundance or richness) and increase *β*-diversity by randomly consuming prey^24^. Specialist predators, which can filter out prey species that are intolerant of them, are predicted to reduce both *α*- and *β*-diversity^24^. The different effects of generalist and specialist predators correspond to the effects of stochastic and deterministic processes in the community assembly, respectively. Predator selectivity within a given pool of prey species theoretically plays a role in the prey community assembly, as well as the degree of CEs on the total prey community size. An empirical determination of how a local community assembly from a regional species pool is influenced by a predator is therefore a fundamental goal of community ecology, because predators can shift the patterns of prey coexistence, abundance, and diversity.

Freshwater comprises only 0.01% and 2.3% of the water on Earth and the global land surface area, respectively^19^, but the number of fish species in freshwater ecosystems is similar to that in marine systems^25^. Recent studies have shown that freshwater organisms suffer from rapid population decline and high extinction risks^26,27^. Nevertheless, the majority of conservation literature remains biased toward terrestrial organisms, with less than 20% of recent studies focusing on aquatic species^28^. Many potential threats to freshwater biodiversity have been evaluated^27^, but the effects of predators on aquatic biodiversity have received less attention, which makes it difficult to understand how aquatic species maintain diversity^29^. Streams with only apex predator (*Lutra lutra*) in taiga forest systems represent an ideal model that can be used to examine the influence of predators on the prey community in natural systems. In this study, we conducted a field investigation to examine the influence of the presence of *L. lutra* on the diversity of fish communities in streams of the Hunchun River Basin, Jilin Province, China. Our study has three objectives: (1) Does the presence of *L. lutra* reduce the abundance of fish and increase the richness of fish species? (2) Does the presence of *L. lutra* increase *β*-diversity of the fish community in stream ecosystems? (3) Does the presence of *L. lutra* mediate the community assembly of fish by increasing the importance of stochastic or deterministic processes?

## Materials and methods

### Study area

**Figure 1:**
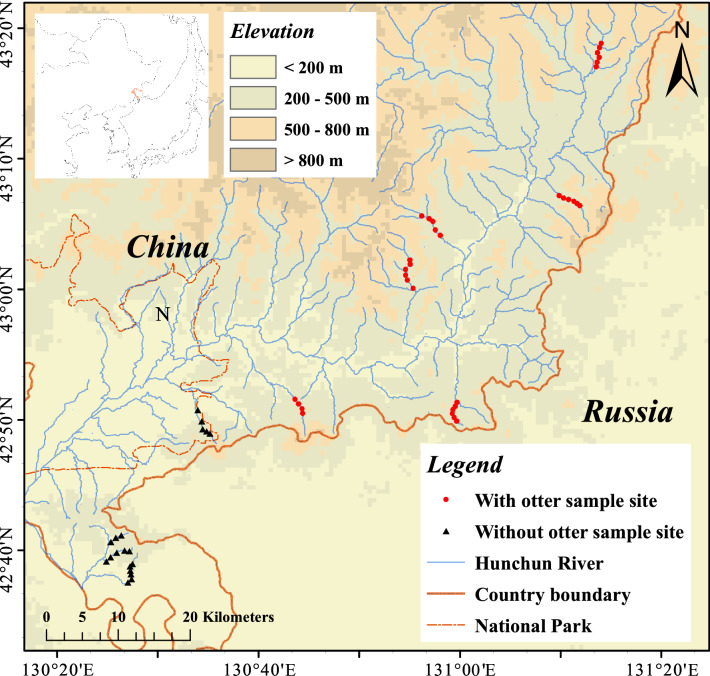
Location of study area within the Hunchun River Basin (HRB), Jilin Province, China. The red circle and black triangle points represented the presence and absence of *L. lutra*, respectively. Map was created using ArcGIS software by Esri (Environmental Systems Resource Institute, ArcGIS 10.8; https://www.esri.com/en-us/home).

The field study was conducted in the Northeast Tiger and Leopard National Park (42° 31′ 06″–44° 14′ 49″ N, 129° 5′ 0″–131° 18′ 48″ E) in the Hunchun River Basin (HRB), Jilin Province, China (Fig. 1). Fish assemblages from the streams were sampled to test the impacts of the presence or absence of *L. lutra* on fish biodiversity. We established transects along 10 streams in the southern area of the HRB (Fig. 1). Each stream was approximately 6 km long. The presence or absence of *L. lutra* in each stream was determined by (1) the presence or absence of *L. lutra* fecal samples collected monthly along each transect from June to September 2021 and (2) camera trapping data from the Tiger-Leopard Observation Network in China^30^, both of methods are widely used in the population investigation of *L. lutra*^31–33^.

### Data collection

We selected 4–6 sample sites for each transect from August to September 2021 and used the collected samples to investigate the compositions of the fish populations at these sites. Each sampling site covered an area with an upstream–downstream length of 200 m with a spacing of least 800 m. We measured the width, depth, flow, bottom quality of the stream, and vegetation cover (50 m around the stream) at 100-m intervals to obtain three sets of data regarding the stream environment at each sample site. Electrofishing was applied to collect fish at each sample site over a 0.5-h period. Each sample species was identified by referring to the relevant reference books and Fish Base Search (www.fishbase.se/home.htm)^34^. For each sample site, we recorded the number of each fish species after identification, and then measured the maximum length and weight of the dominant species (30% of the total number). All samples were released after data collection.

The diversity of the fish assemblages was specifically represented by the species richness, Shannon–Wiener index, species evenness, catch per unit effort (CPUE, in 1-h period), and the CPUE of the dominant species. The Shannon–Wiener indices were calculated for each sample site according to (1):

1 $$\begin{array}{*{20}c} {H = - \mathop \sum \limits_{i = 1}^{n} xi\log_{2} xi} \\ \end{array}$$

where *H* is the Shannon–Wiener index, *xi* represents the proportion of the *i*-th species to the total, and *n* is the number of fish species. The Shannon–Wiener index was calculated using the Past4 Project 1.0.0.0 (Past 4—the Past of the Future—Natural History Museum (uio.no)).

The species evenness was calculated according to (2):

2 $$\begin{array}{*{20}c} {E = H/H_{max} } \\ \end{array}$$

where *E* is species evenness, *H* is the Shannon–Wiener index, $H_{max}$ is the maximum Shannon–Wiener index to the total. The species evenness indices were also calculated using the Past4 Project 1.0.0.0 (Past 4—the Past of the Future—Natural History Museum (uio.no)).

We also calculated the $\beta$*-*diversity index to reflect the impact of *L. lutra* on the fish communities in each sample sites according to (3):

3 $$\begin{array}{*{20}c} {\beta = 1 - \frac{2z}{{x + y}}} \\ \end{array}$$

where *x* and *y* are the abundances of fish species in the first and second fish communities, respectively, and *z* is the minimum abundance of the common species to both communities. The *β-*diversity indices were calculated using the R package functions ‘*vegan*’ and ‘*reshape2*’.

The sampled fish species were assigned to functional categories based on diet (Nutritional Functional Group), morphological traits (Morphological Functional Group), habitat (Habitat Functional Group), and behavior (Reproductive Functional Group), as described in Fish Base Search (www.fishbase.se/home.htm).

### Statistical analyses

Linear mixed models (LMMs) were fitted to model the influence of *L. lutra* on the fish communities. The Shannon–Wiener index, species evenness, species richness, CPUE, *β*-diversity, abundance of the fish functional groups, and the CPUE of the dominant species were the response variables. The habitat variables of the streams, including the width, depth, flow, bottom quality of the stream, and vegetation cover were considered as random effects in the models to distinguish between the effects of non-independent variables. The presence (1) and absence (0) of *L. lutra* in each sample site was included in the model as a fixed explanatory effect. The models were applied using SPSS (IBM SPSS Statistics 25. Ink SPSS Software|IBM).

### Statement for use of experimental animals

The applied fish sampling protocols and methods were congruent with the institutional guidelines of the Dalian Ocean University. Samples and data were collected according to the regulations of Ethic and Animal Welfare Committee College of Life Science Beijing Normal University. All experimental protocols were approved by Dalian Ocean University Animal Care and Use Committee (DLOU-ACUC-20210524). The reporting in the manuscript follows the recommendations in the ARRIVE guidelines (https://arriveguidelines.org/).

## Results

Among the 10 sampled streams, six contained *L. lutra* and four did not (Fig. 1). A total of nine fish species were observed, including *Phoxinus lagowskii*, *Phoxinus phoxinustumensis*, *Barbatula barbatula nuda*, *Lefua costata*, *Cottus poecilopus* Heckel, *Salvelinus malma*, *Oncorhynchus masou*, *Pungitius sinensis*, and *Lampetra reissneri* Dybowski (Table 1). Only *P. lagowskii* appeared in all of the sampled streams as a dominant species (Table 1).

**Table 1:** The fish species were found in the Hunchun River Basin.

	*Phoxinus lagowskii*	*Phoxinus phoxinustumensis*	*Cottus poecilopus* Heckel	*Barbatula barbatula nuda*	*Lefua costata*	*Salvelinus malma*	*Oncorhynchus masou*	*Pungitius sinensis*	*Lampetra reissneri* Dybowski
N1	**+**	−	**−**	**+**	**+**	**−**	**−**	**−**	**−**
N2	**+**	**−**	**+**	**+**	**+**	**−**	**−**	**+**	**−**
N3	**+**	**−**	**−**	**+**	**+**	**−**	**−**	**+**	**−**
N4	**+**	**−**	**−**	**+**	**−**	**−**	**−**	**−**	**−**
Y1	**+**	**+**	**+**	**+**	**−**	**−**	**−**	**−**	**−**
Y2	**+**	**+**	**+**	**+**	**−**	**+**	**+**	**−**	**−**
Y3	**+**	**−**	**+**	**+**	**−**	**−**	**−**	**−**	**−**
Y4	**+**	**−**	**+**	**+**	**−**	**+**	**−**	**+**	**+**
Y5	**+**	**+**	**+**	**+**	**−**	**−**	**−**	**−**	**−**
Y6	**+**	**+**	**+**	**+**	**−**	**+**	**−**	**+**	**−**

N_i_, stream without otter; Y_i_, stream with otter; + /**−**, presence/absence of fish species.

**Figure 2:**
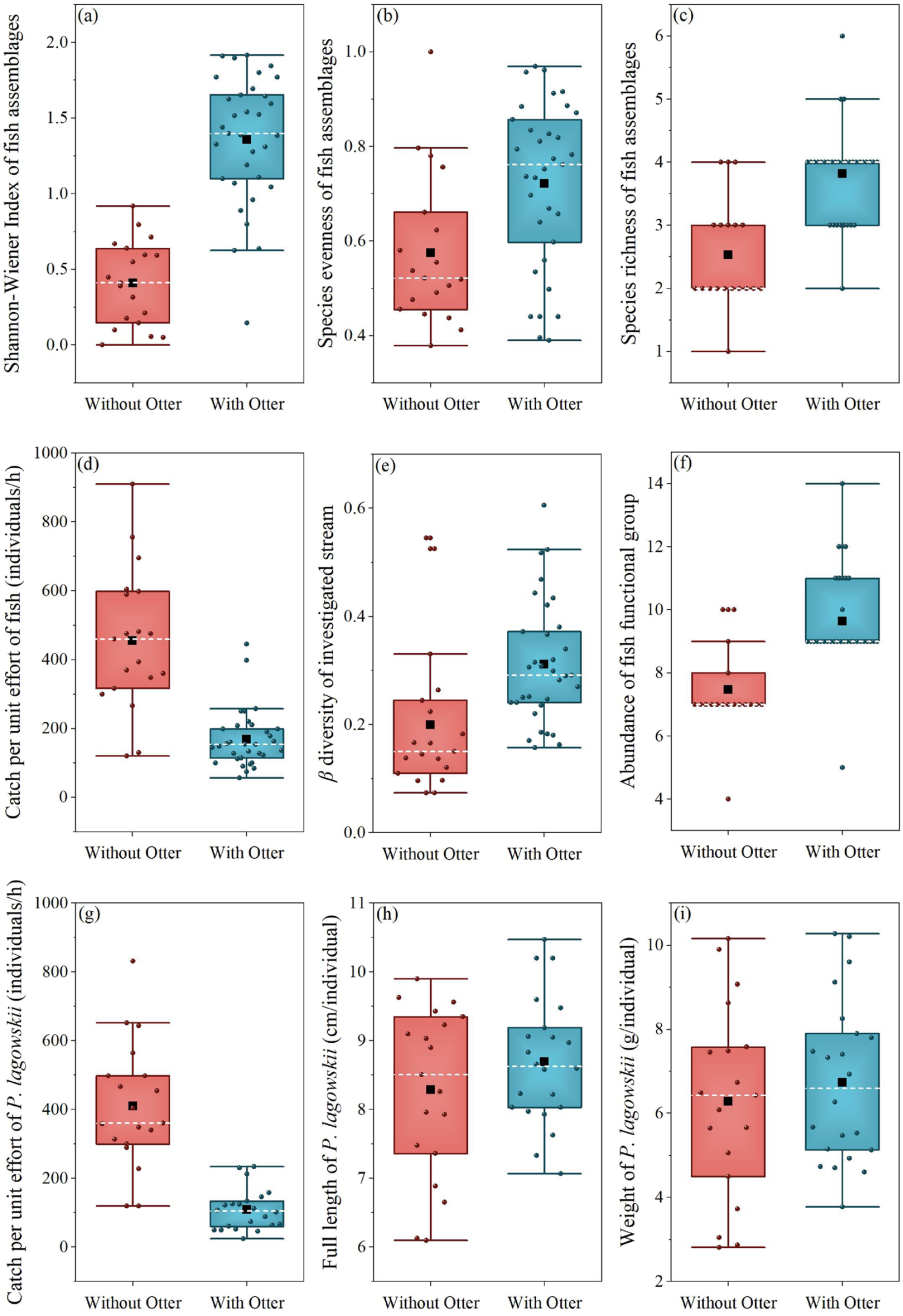
Box whisker plots of the diversity of fish assemblages in the presence and absence of *L. lutra*. Each picture shows the difference in fish assemblages with or without *L. lutra*. The white dotted line is median and the black square is the mean. The deviation bars represent the 5th and 95th percentiles.

The presence of *L*. *lutra* was associated with a significantly higher diversity of fish assemblages in the streams (LMM _Shannon–Wiener Index_, *d.f*1 = 1, *d.f*2 = 8.349, *F* = 28.427, *P* = 0.001; LMM _species richness_, *d.f*1 = 1, *d.f*2 = 7.735, *F* = 14.178, *P* = 0.006) and *β*-diversity (LMM _*β*-diversity_, *d.f*1 = 1, *d.f*2 = 7.882, *F* = 7.673, *P* = 0.025) (Table 2, Fig. 2a,c,e). In contrast, the species evenness, full length and weight of *P. lagowskii* (dominant species) was not significantly influenced by *L. lutra* (LMM _species evenness_, *d.f*1 = 1, *d.f*2 = 8.348, *F* = 3.166, *P* = 0.112) (Table 2, Fig. 2b,h,i). The absence of *L*. *lutra* also had a significant impact on the CPUE (1 h) (LMM _CPUE_, *d.f*1 = 1, *d.f*2 = 8.075, *F* = 15.472, *P* = 0.004; LMM _CPUE of dominant species_, *d.f*1 = 1, *d.f*2 = 8.037, *F* = 26.104, *P* = 0.001) (Table 2, Fig. 2d,g). The number of functional groups was higher in the streams with *L. lutra* than in the streams without *L. lutra* (LMM _functional group_, *d.f*1 = 1, *d.f*2 = 8.132, *F* = 10.038, *P* = 0.013) (Table 3, Fig. 2f).

**Table 2:** The effects of otter presence/absence on the fish assemblages in the Hunchun River Basin from Liner Mix Models (LMMs).

	*d.f*1	*d.f*2	*F*	*P*
Shannon–Wiener Index	1	8.349	28.427	0.001
Species evenness	1	8.348	3.166	0.112
Species richness	1	7.735	14.178	0.006
CPUE of fish	1	8.075	15.472	0.004
*β* diversity	1	7.882	7.673	0.025
Abundance of fish functional group	1	8.132	10.038	0.013
CPUE of Dominant Species	1	8.037	26.104	0.001

CPUE = Catch Per Unit Effort (1 h); Dominant Species: *P.lagowskii; d.f.* = degrees of freedom, *F* = *F*-ratio.

**Table 3:** The functional groups of fish assemblages in the Hunchun River Basin.

species	Nutritional Functional group	Morphological Functional group	Habitat Functional group	Reproductive Functional group
*Phoxinus lagowskii*	Insects	Weak spindle	Torrent swim	No-Protagonist Sticky egg
*Phoxinus phoxinustumensis*	Insects	Weak spindle	Torrent swim	No-Protagonist Sticky egg
*Barbatula barbatula nuda*	Insects/Vegetative	Cylindrical	Slow flow benthic	No-Protagonist Sticky egg
*Lefua costata*	Insects/Vegetative	Cylindrical	Slow flow benthic	No-Protagonist Sticky egg
*Cottus poecilopus* Heckel	Insects/Fish/Crustaceans	Cylindrical	Slow flow benthic	Protagonist Sticky egg
*Salvelinus malma*	Insects/Fish/Crustaceans	Flat	Torrent swim	No-Protagonist Sinking egg
*Oncorhynchus masou*	Insects/Fish/Crustaceans	Strong spindle	Torrent swim	No-Protagonist Sinking egg
*Pungitius sinensis*	Zooplankton	Weak spindle	Still water swim	Protagonist Floating eggs
*Lampetra reissneri* Dybowski	Zooplankton/Vegetative/parasitism	Cylindrical	Slow flow benthic	No-Protagonist Sticky egg

## Discussion

In this study, *L*. *lutra* is investigated as a potential umbrella species in a stream ecosystem^35–37^, whose presence naturally promotes the coexistence of a large number of fish taxa. The interaction of *L*. *lutra* and the fish community is an empirical example of the role of top predators in regulating prey biodiversity, and underscores the importance of top-down control in ecological communities. As a generalist predator, *L*. *lutra* was associated with a reduced abundance but enhanced richness of fish assemblages, both of which could enhance the fish community diversity. The *β*-diversity was also found to increase in the presence of *L*. *lutra* due to the enhanced fish species richness. We suggest that *L*. *lutra* likely regulated the fish community assembly through the stochastic process.

Predator effects on the biomass and biodiversity of prey species are well understood^38^ and also verified in this study. Differing from theoretical and empirical observations^20,24^, *L*. *lutra* as a generalist predator was found in this study to enhance the *α*-diversity of fish assemblages (e.g., Shannon–Wiener index and species richness). This indicates that the presence of *L. lutra* not only influenced the number of fish species, but also changed the uniformity of fish species in the community. The diet of *L*. *lutra* in this region mainly comprises stream fishes, of which *Phoxinus*, *Cottus*, and *Cobitis* make the highest contribution (unpublished data); these were also the dominant fish species of the stream ecosystem in this region. Therefore, when the stream fishes are under the predation of *L. lutra*, the number of the most dominant species (*Phoxinus*) decreases, whereas the uniformity of the fish species in the community would be enhanced, thus resulting in a higher Shannon–Wiener index. Compared with specialist predators, generalist predators may not completely remove a prey species at the local scale through CEs; as a result, generalist predators may alter the interspecific competition between prey species through NCEs and enhance the species richness of the community^39,40^.

Although our study does not explicitly elucidate the mechanisms of stochastic process in shaping the fish community assembly, we speculate that the increasing *β*-diversity of the fish community through stochastic process primarily results from the presence of *L. lutra* in the studied streams. Predators can either strengthen stochastic process and increase *β*-diversity by reducing the population size of all prey populations, or they can strengthen deterministic process leading to species extinction, or both processes can simultaneously occur in structuring the community assembly^20,41,42^. In our study, the presence of *L. lutra* was associated with a reduced abundance of dominant fish species and an overall abundance of fish, but clearly showed positive impacts on several rare fish species in the study area (e.g., *S*. *malma*, *O. masou*, *L*. *reissneri*). Both of these processes could enhance the overall fish richness and species heterogeneity in different rivers, which may contribute to higher *β*-diversity. The habitat variables of the streams also showed no impact on fish community diversity. These lines of evidence support that the stochastic process predominates in the community assembly of fish species. To verify the coexistence mechanisms of the fish species, further studies should be designed in association with the phenotypic plasticity^43^ and physiological response^44^ in response to CEs or NCEs.

Functional groups are sensitive to changes in species richness, especially the functional groups of species at higher trophic levels^45,46^. Our results indicate that the top-down effects of *L. lutra* could enhance the functional diversity of stream fishes, which may be due to the regulation of the predator on the prey species richness^39^. In this study, three rare species (e.g., *S*. *malma*. *O. masou*, *L*. *reissneri*) provided four unique functional groups, such as parasitism of the nutritional functional group, a flat and strong spindle of the morphological functional group, and a no-protagonist sinking egg of the reproductive functional group, all of which increased the functional groups owing to the higher species richness. Rare species often provide unique functional contributions, thus the loss of functional diversity may constrain the community and ecosystem processes^47^.

## Conclusion

Our results show that the presence of *L. lutra* influenced the variations of taxonomic and functional diversity of fishes in streams of a taiga forest system. This work suggests that this predator can increase the importance of stochastic process in shaping the fish community assembly by reducing the size of all prey populations. This study also provides empirical field results that emphasizes the role of predators in maintaining biodiversity and ecosystem function. However, both of the interactions of different fish species and unique habitat selection of carnivorous fish species may simultaneously impact the fish community, except the impacts of *L. lutra.* As a pilot and preliminary research, our results help the understanding of predation on prey communities. If the impacts of multi-predator interactions on prey communities needed to be investigate, greater sampling efforts will be possible in the future. Future conservation measures involving biodiversity in ecosystems should require the inclusion of predation strength. The inclusion of scientific research and protection of umbrella species would thus constitute an additional and important step in biodiversity conservation.

## Data Availability

The datasets are available in the following repository [https://doi.org/10.5061/dryad.fqz612jvt]. Link: [https://datadryad.org/stash/share/qDAkFKfwCI-JTok3qKs4VAYbzzr_7mpo8Qdix8Al-0Q].
